# Prognostic significance of pre-treatment neutrophil-to-lymphocyte ratio (NLR) in patients with oropharyngeal cancer treated with radiotherapy

**DOI:** 10.1038/s41416-020-01106-x

**Published:** 2020-10-14

**Authors:** Sweet Ping Ng, Houda Bahig, Amit Jethanandani, Erich M. Sturgis, Faye M. Johnson, Baher Elgohari, G. Brandon Gunn, Renata Ferrarotto, Jack Phan, David I. Rosenthal, Steven J. Frank, Clifton D. Fuller, Adam S. Garden

**Affiliations:** 1 Department of Radiation Oncology, The University of Texas MD Anderson Cancer Center, Houston, TX USA; 2 Department of Radiation Oncology, Peter MacCallum Cancer Centre, Melbourne, VIC Australia; 3 Department of Radiation Oncology, Centre Hospitalier de l’Universite de Montreal, Montreal, QC Canada; 4 Department of Head and Neck Surgery, The University of Texas MD Anderson Cancer Center, Houston, TX USA; 5 Department of Thoracic Head and Neck Medical Oncology, The University of Texas MD Anderson Cancer Center, Houston, TX USA

**Keywords:** Prognostic markers, Head and neck cancer

## Abstract

**Background:**

This study aimed to evaluate the prognostic value of pre-treatment NLR in patients with oropharyngeal cancer.

**Methods:**

Patients who completed definitive radiotherapy (RT) for oropharyngeal cancer and had blood counts taken pre-RT from 2002 to 2013 were included. NLR was calculated as total neutrophil/lymphocytes. Survival rates were estimated using the Kaplan–Meier method. Univariable and multivariable analyses were conducted with linear and Cox regression methods. NLR was analysed posteriori and dichotomised on the discovered median.

**Results:**

Eight hundred and forty-eight patients were analysed. The median pre-RT NLR was 3. Patients with NLR of <3 had improved overall survival (OS) than those with NLR ≥ 3 (5-year OS 85 vs 74%, *p* < 0.0001). OS differences remained significant when stratified according to HPV status (HPV-positive *p* = 0.011; HPV-negative *p* = 0.003). Freedom from any recurrence (FFR), locoregional control (LRC) and freedom of distant recurrence (FDR) were better in those with NLR < 3. The negative impact of elevated pre-RT NLR on OS (HR = 1.64, *p* = 0.001), FFR (HR = 1.6, *p* = 0.006) and LRC (HR = 1.8, *p* = 0.005) remained significant on multivariable analysis.

**Conclusions:**

Pre-RT NLR is an independent prognostic factor in patients with oropharyngeal cancer regardless of HPV status. Patients with lower NLR had more favourable OS and disease control.

## Background

The incidence of oropharyngeal cancer is on the rise in the developed countries.^1^ As human papillomavirus (HPV) is an infectious agent, there is renewed interest to investigate the extent of host’s inflammatory reaction to the persistent HPV infection contributing to neoplastic transformation, cancer treatment response and patient’s prognosis. While inflammation can be protective against malignancy in the initial phase, activating the innate immune system and recruiting primitive immune cells such as neutrophils to the site is attributed to promote tumorigenesis and cancer progression.^2^ It is postulated that chronic inflammation promotes quick turnover of cells, thereby accumulating and propagating mutations contributing to malignant transformation. In addition, the inflammatory cascade leads to capillary leakiness potentially promoting tumour angiogenesis and metastatic potential.

Neutrophil-to-lymphocyte ratio (NLR) is a simple biomarker of systemic inflammation and has been demonstrated to be a prognostic marker in several solid cancers, including prostate,^3^ renal,^4^ gastric,^5^ brain^6^ and hypopharyngeal^7^ cancers. Here we evaluated the effect of pre-treatment NLR on outcomes in patients with oropharyngeal cancer in the contemporary era.

## Methods

This retrospective study was approved by the Institutional Review Board of The University of Texas MD Anderson Cancer Center. This study was performed in accordance with the Declaration of Helsinki. All patients, above the age of 18 years, who completed curative-intent radiotherapy for squamous cell carcinoma of the oropharynx and had blood counts taken within 2 weeks before radiotherapy from 2002 to 2013 were included in this study. All patients received curative-intent radiation dose. Patients with distant metastatic disease (M1) at diagnosis, had no blood counts taken within 2 weeks of commencing radiotherapy or had a haematologic disorder affecting lymphocyte and/or neutrophil counts were excluded. Patient, tumour and treatment characteristics, clinical outcomes and pre-radiotherapy total neutrophil and lymphocyte counts (TNC and TLC, respectively) were recorded. HPV status was collected, whenever available, and is deemed positive if either p16 immunohistochemistry or HPV in situ hybridisation was positive. The disease was staged according to the American Joint Committee on Cancer (AJCC) staging system (Seventh edition). NLR was calculated as TNC divided by TLC. NLR was analysed posteriori and dichotomised on the discovered median (rounded to nearest whole number).

### Statistical analysis

Overall survival (OS) was calculated with the Kaplan–Meier method from the date of completion of radiotherapy to date of death. Freedom from locoregional failure was measured from the date of completion of radiotherapy to the date of first locoregional failure. Freedom from distant metastasis was calculated from the date of completion of radiotherapy to the date of first distant disease. Freedom from recurrence was calculated from the date of completion of radiotherapy to the date of any first recurrence. For all survival calculations, patients without events were censored at last follow-up time.

The impact of NLR on survival and disease control rates was estimated using the Kaplan–Meier method and compared with log-rank tests. Potential prognostic factors for OS and freedom from recurrence were evaluated with univariable and multivariable analyses and were conducted with linear and Cox proportional hazard regression models. Variables that achieved a *p* value of ≤0.1 in univariable analyses were included in the multivariable analysis. A two-tailed *p* value of <0.05 was deemed statistically significant. Statistical analyses were performed using JMP v14.0 (SAS Institute Inc.).

## Results

### Patient characteristics

From 2002 to 2013, 1124 patients with localised oropharyngeal cancer received definitive radiotherapy. Of these, 276 patients were excluded from this analysis: 273 patients did not have a blood count within 2 weeks of commencing radiotherapy, 2 had chronic lymphocytic leukaemia, and 1 had a spurious high neutrophil count. Therefore, a total of 848 patients were eligible for analysis. Table 1 summarises the patient, disease and treatment characteristics. The median age of the cohort was 57 years (range: 29–87 years). The majority (87%) were males and approximately half the cohort were never smokers or previous smokers with <10 pack year history. Base of tongue and tonsil were the predominant sites accounting for 98% of the cohort primary site. Six hundred and three (71%) patients had HPV/p16-positive squamous cell carcinoma. The median radiation dose fractionation delivered was 70 Gy in 33 fractions. Almost half the cohort received induction chemotherapy and 88% had concurrent chemotherapy.

**Table 1:** Patient, disease and treatment characteristics.

Parameters	Cohort (*n* = 848)	Percentage (%)
Age (median)	57 years (range: 29–87)	
Sex		
Male	741	87.4
Female	107	12.6
Smoking status		
Never	364	42.9
Previous <10 pack years	97	11.4
Previous >10 pack years	215	25.3
Current	172	20.4
Primary site		
Base of tongue	463	54.7
Tonsil	368	43.4
Soft palate	10	1.2
Pharyngeal wall	7	0.8
Tumour (T) stage^a^		
T1	155	18.2
T2	304	35.9
T3	209	24.6
T4	162	19.1
Tx	18	2.1
Nodal (N) stage^a^		
N0	41	4.8
N1	74	8.7
N2a	51	6.0
N2b	423	49.8
N2c	211	25.0
N3	45	5.3
Nx	3	0.4
Stage (AJCC Seventh edition)^a^		
II	11	1.3
III	82	9.7
IV	755	89.0
HPV/p16 status		
Positive	602	71.0
Negative	71	8.4
Unknown	175	20.6
Dose (median)	6996 cGy (range: 4800–7396)	
Number of fractions (median)	33 (range: 28–42)	
Induction chemotherapy		
Yes	382	45.1
No	466	54.9
Concurrent chemotherapy		
Yes	742	87.5
No	106	12.5

^a^Tumours were staged according to the AJCC Seventh edition.

### Pre-treatment NLR and outcomes

The median pre-treatment NLR was 2.52 (range: 0.05–23.9). The median follow-up time was 59 months (range: 6–153 months). At last follow-up, 183 (22%) patients had died. Overall, 141 (17%) patients developed disease recurrence: 70 with distant disease (including 11 with local and/or regional disease, 37 with local disease, 29 with regional disease, and 5 with local and regional disease.

**Fig. 1:**
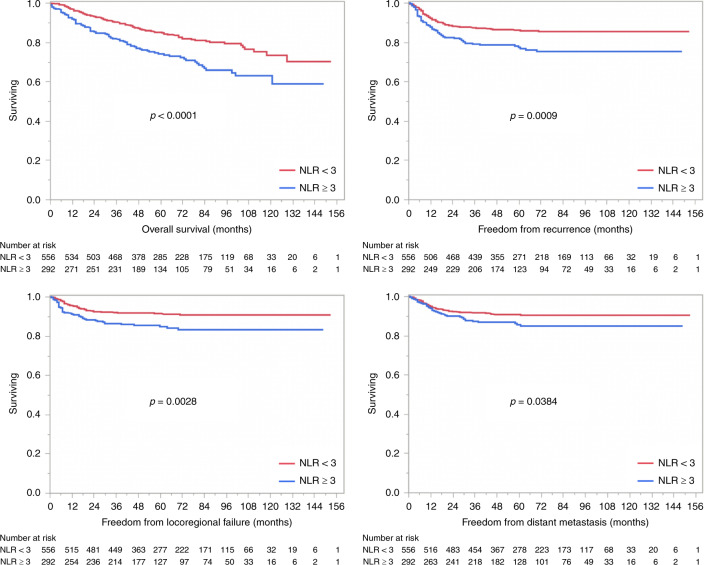
Overall survival, freedom from recurrence, locoregional control, and distant metastasis for the cohort.

Patients with NLR < 3 had a 5-year OS of 85% compared to 74% (*p* < 0.0001) for those with NLR ≥ 3. Freedom from recurrence, locoregional failure and distant metastasis were better in those with NLR < 3 (5-year freedom from recurrence 86 vs 77%, *p* = 0.0009; 5-year freedom from locoregional failure 92 vs 85%, *p* = 0.003; 5-year freedom from distant metastasis 91 vs 86%, *p* = 0.038; Fig. 1). To verify that our results were not influenced by outliers, we re-analysed the data with just the patients whose NLR was in the 5–95% values (range: 0.95–7.97). This demonstrated that the improved outcomes in those with NLR < 3, as shown in the overall cohort analysis, remained significant.

### Stratification by HPV status

**Fig. 2:**
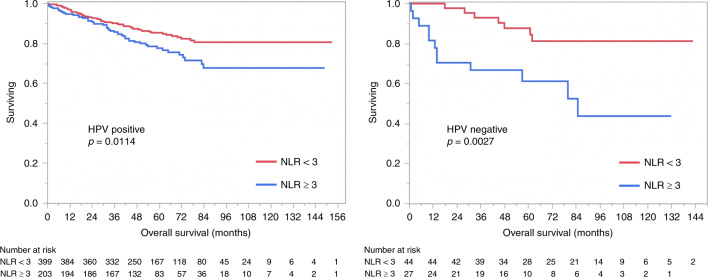
Overall survival according to HPV/p16 status.

**Fig. 3:**
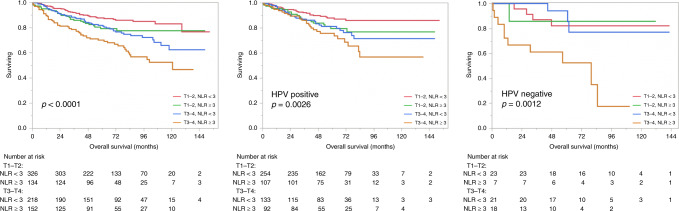
Overall survival stratified according to tumour (T) stage and NLR.

HPV status was available for 674 patients—603 HPV positive and 71 HPV negative. When stratified according to HPV status, those with NLR < 3 continued to have a significantly better OS than those with NLR ≥ 3 (HPV positive: 5-year OS 85 vs 78%, *p* = 0.011; HPV negative: 5-year OS 88 vs 61%, *p* = 0.003; Fig. 2). Similar effect is observed for freedom from recurrence (HPV positive: 5-year 86 vs 80%, *p* = 0.036; HPV negative: 5-year 84 vs 69%, *p* = 0.051). On logistic regression analysis, there was association between NLR and T stage (*p* = 0.0003) and N stage (*p* = 0.002) but no significant association with HPV status (*p* = 0.75) nor smoking status (*p* = 0.88). Figure 3 shows the impact of combined T stage and NLR on OS.

### Univariable and multivariable analyses

Univariable analyses of variable of interest are shown in Table 2. On multivariable analyses, NLR remained as an independent prognostic factor for OS and freedom from recurrence with NLR ≥ 3 having a risk ratio of 1.64 (95% confidence interval (CI) 1.22–2.19, *p* = 0.001) and 1.62 (95% CI 1.15–2.26, *p* = 0.006), respectively. In addition to NLR, patient’s smoking status, age and T stage were also associated with OS outcome. Radiation dose (higher dose) and NLR < 3 were associated with improved freedom from recurrence.

**Table 2:** Univariable and multivariable analyses of prognostic factors associated with outcomes.

Parameters	Comparator	Reference	Overall survival		Freedom from recurrence		Freedom from locoregional failure		Freedom from distant metastasis	
			Univariable (HR, 95% CI)	Multivariable (HR, 95% CI)	Univariable (HR, 95% CI)	Multivariable (HR, 95% CI)	Univariable (HR, 95% CI)	Multivariable (HR, 95% CI)	Univariable (HR, 95% CI)	Multivariable (HR, 95% CI)
Age (per unit change)			<0.0001* (1.03, 1.01–1.05)	0.003 (1.02, 1.01–1.04)	0.095* (1.02, 1.00–1.03)	0.424 (1.01, 0.99–1.03)	0.159 (1.02, 0.99–1.04)		0.122 (1.02, 1.00–1.04)	
Sex	Female	Male	0.144 (0.71, 0.42–1,12)		0.416 (0.81, 0.45–1.33)		0.633 (0.85, 0.42–1.57)		0.121 (0.57, 0.24–1.14)	
Smoking status	Never/Ex <10	Current/Ex >10	0.0001* (0.57, 0.42–0.76)	0.002 (0.63, 0.47–0.85)	0.015* (0.67, 0.47–0.92)	0.068 (0.73, 0.52–1.02)	0.005* (0.55, 0.36–0.84)	0.021 (0.61, 0.40–0.93)	0.530 (0.88, 0.58–1.33)	
HPV status	Negative/ Unknown	Positive	0.193 (1.34, 0.80–2.14)		0.325 (1.20, 0.83–1.69)		0.658 (1.11, 0.70–1.72)		0.787 (1.06, 0.67–1.66)	
T stage	Tx–T2	T3–T4	<0.0001* (0.52, 0.38–0.69)	0.005 (0.62, 0.43–0.86)	0.001* (0.57, 0.40–0.79)	0.361 (0.84, 0.58–1.21)	0.001* (0.50, 0.33–0.76)	0.368 (0.81, 0.51–1.27)	0.092* (0.70, 0.46–1.06)	0.886 (1.03, 0.66–1.60)
N stage	N0–N2a	N2b–N3	0.197 (0.78, 0.52–1.13)		0.674 (0.91, 0.58–1.37)		0.845 (0.95, 0.54–1.57)		0.798 (0.93, 0.53–1.54)	
AJCC stage	I–II	III–IV	0.949 (0.96, 0.16–2.99)		0.066* (2.02^e−9^, 1.14–1.14)	0.120 (4.861^e−11^, 8.14^e−243^–1.60)	0.147 (2.02 ^e−9^, 1.84–1.84)		0.145 (2.02^e−9^, 1.82–1.82)	
Induction	Yes	No	0.216 (1.20, 0.90–1.61)		0.114 (1.31, 0.94–1.82)		0.189 (1.32, 0.87–2.01)		0.197 (1.31, 0.87–2.00)	
Concurrent	Yes	No	0.017* (1.84, 1.11–3.32)	0.38	0.027* (1.93, 1.07–3.93)	0.502 (1.25, 0.67–2.62)	0.002* (4.26, 1.60–17.35)	0.060 (2.67, 0.97–11.10)	0.135 (1.72, 0.86–4.11)	
Radiation dose (per unit change)			0.006* (1.001, 1.00–1.002)	0.77	<0.0001* (1.002, 1.001–1.003)	0.004 (1.001, 1.001–1.003)	<0.0001* (1.002, 1.001–1.004)	0.009 (1.001, 1.000–1.004)	<0.0001* (1.002, 1.001–1.004)	0.0003 (1.002, 1.001–1.004)
NLR	≥3	<3	<0.0001* (1.81, 1.35–2.42)	0.001 (1.64, 1.22–2.19)	0.002* (1.72, 1.23–2.39)	0.006 (1.62, 1.15–2.26)	0.005* (1.82, 1.20–2.76)	0.010 (1.75, 1.14–2.66)	0.041* (1.56, 1.02–2.36)	0.052 (1.52, 0.99–2.32)

*HPV* human papillomavirus, *T* tumour, *N* nodal, *AJCC* American Joint Committee on Cancer, *NLR* neutrophil-to-lymphocyte ratio, *HR* hazard ratio, *CI* confidence interval.

Results are reported in *p* values. Parameters that had a *p* value of <0.10 in univariable analysis were included in multivariable analysis and are denoted by asterisk (*).

To determine whether the effect of NLR is relatively linear as an overall continuous degree of inflammation, NLR was analysed as a continuous variable. On univariable analysis, lower NLR was associated with better freedom from recurrence (*p* = 0.003, HR = 1.04, 95% CI = 1.01–1.06) and locoregional failure (*p* = 0.007, HR = 1.05, 95% CI = 1.01–1.07). However, NLR as a continuous variable had no significant correlation with OS (*p* = 0.37) and freedom from distant metastasis (*p* = 0.92).

## Discussion

Our study which consisted of a large cohort of patients with squamous cell carcinoma of the oropharynx demonstrated that the pre-radiotherapy NLR has significant impact on OS and disease control. Patients with NLR of <3 before radiotherapy had an improved 5-year survival of 85% compared to 74% in those with NLR of 3. The impact of NLR on clinical outcomes was independent of HPV status.

NLR is a simple inflammatory marker that has been proven to be a prognostic marker in multiple malignancies.^3–6,8^ A large meta-analysis reporting on a 100 studies with >40,000 patients demonstrated that patients with solid tumours and a higher NLR (>4) had worse OS and disease outcomes, regardless of cancer stage or subsites.^8^ In head and neck cancer, NLR has been extensively investigated as a prognostic marker although variable cut-off values and timepoints were used.^7,9–15^ The majority of studies have included a heterogeneous group of patients with tumours from differing head and neck subsites.^7,11,13–16^ More recently, in a smaller study than ours, So et al.^12^ reported on a cohort of 104 patients with HPV-associated oropharyngeal cancer and showed that patients with high NLR had worse 5-year disease-free survival. In patients with HPV-negative disease, Lin et al.^17^ reported that an elevated NLR at 3 months after completion of radiotherapy was associated with worse survival. Our study results are consistent with previous literature and provide a validation for the use of pre-treatment NLR as a prognostic marker in a contemporary cohort of patients with oropharyngeal cancer.

The relationship between inflammation and cancer has become an increasingly interesting but intricate area of research. Although inflammation has been identified as one of the hallmarks of cancer,^18^ the complex relationship between inflammation and the tumour microenvironment, promoting angiogenesis and malignant transformation and subsequently cancer progression, remain poorly understood. While HPV is now identified as a cause of oropharyngeal cancer, our study has shown that NLR remained as an effective prognostic biomarker regardless of viral status. One might assume that the persistent HPV infection releases pro-inflammatory cytokines resulting in chronic inflammation and subsequently carcinogenesis. However, it appears that the inflammatory tumour microenvironment may differ between HPV-positive and HPV-negative tumours, as patients with HPV-positive oropharyngeal cancer have improved outcomes compared to those with HPV-negative cancer. For example, it has been noted that patients with HPV-positive oropharyngeal cancer tend to have radiologically cystic or necrotic neck nodes^19,20^ compared to those with HPV-negative disease. Necrosis is proinflammatory and can recruit immune cells to the area with the intent of clearing the necrotic debris.^18^ Although the intent of inflammatory infiltrate is to remove debris and promote healing, interleukin-1α released by necrotic cells for cell proliferation can inadvertently expedite neoplastic transformation and progression.^21^

Our study comes with caveats of a single institution retrospective cohort study. Second, relative to those with HPV-positive disease, the number of patients with HPV-negative oropharyngeal cancer in this cohort is only 71 (8.4%), thereby limiting the statistical power and further analysis to determine the impact of NLR on disease-specific outcomes due to the small number of events. Third, although we reported the number of patients who received induction and concurrent chemotherapy, we did not detail the chemotherapeutic agents. The vast majority who received induction chemotherapy received taxane–platinum-based regimens, while our concurrent patients received single agent platin or cetuximab. Nevertheless, our results are consistent with the previous studies in head and neck and other cancers indicating that NLR, as a marker of systemic inflammation, is prognostic for clinical outcomes.

In this large cohort of patients with oropharyngeal cancer, we highlighted that pre-radiotherapy NLR is an independent prognostic factor in patients with oropharyngeal cancer regardless of HPV status. Patients with lower NLR had more favourable clinical outcomes in terms of survival and disease control. NLR could be explored prospectively as a potential cost-effective biomarker for further pre-treatment risk stratification of patients with oropharyngeal cancer for treatment de-escalation/escalation.

## Data Availability

Data will be available on request.
